# Optimal Timing of Simethicone Supplement for Bowel Preparation: A Prospective Randomized Controlled Trial

**DOI:** 10.1155/2021/4032285

**Published:** 2021-10-27

**Authors:** Zhen-wen Wu, Sheng-gang Zhan, Mei-feng Yang, Yi-teng Meng, Feng Xiong, Cheng Wei, Ying-xue Li, Ding-guo Zhang, Zheng-lei Xu, Ben-hua Wu, Rui-yue Shi, Jun Yao, Li-sheng Wang, De-feng Li

**Affiliations:** ^1^Department of Gastroenterology, Shenzhen People's Hospital, (The Second Clinical Medical College, Jinan University), Shenzhen 518020, Guangdong, China; ^2^Department of Gastroenterology, Shenzhen People's Hospital, (The First Affiliated Hospital, Southern University of Science and Technology), Shenzhen 518020, Guangdong, China; ^3^Department of Hematology, Yantian District People's Hospital, Shenzhen 518020, Guangdong, China

## Abstract

**Background and Aims:**

Simethicone (SIM), as an antifoaming agent, has been shown to improve bowel preparation during colonoscopy. However, the optimal timing of SIM addition remained undetermined. We aimed to investigate the optimal timing of SIM addition to polyethylene glycol (PEG) to improve bowel preparation.

**Methods:**

Eligible patients were randomly assigned to two groups: the SIM evening group (SIM addition to PEG in the evening of the day prior to colonoscopy) and the SIM morning group (SIM addition to PEG in the morning of colonoscopy). The primary outcome was Bubble Scale (BS). The secondary outcomes were Boston Bowel Preparation Scale (BBPS) and adenoma detection rate (ADR).

**Results:**

A total of 419 patients were enrolled in this study. The baseline characteristics of the patients were similar in both groups. No significant differences were observed in terms of BS (8.76 ± 0.90 vs. 8.65 ± 1.16, *P* = 0.81), ADR (34.1% vs. 30.8%, *P* = 0.47), Boston Bowel Preparation Scale (BBPS) (8.59 ± 0.94 vs. 8.45 ± 1.00, *P* = 0.15), and withdrawal time (8.22 ± 2.04 vs. 8.01 ± 2.51, *P* = 0.094) between the two groups. Moreover, safety and compliance were similar in both groups. However, the SIM evening group was associated with shorter cecal intubation time (3.80 ± 1.81 vs. 4.42 ± 2.03, *P* < 0.001), higher BS (2.95 ± 0.26 vs. 2.88 ± 0.38, *P* = 0.04) in the right colon, and diminutive ADR (62.5% vs. 38.6%, *P* = 0.022) in the right colon, when compared to the SIM evening group.

**Conclusions:**

The SIM addition to PEG in the evening of the day prior to colonoscopy can shorten cecal intubation time and improve BS scores and diminutive ADR of the right colon compared with the SIM addition to PEG in the morning of colonoscopy in bowel preparation.

## 1. Introduction

Colorectal cancer (CRC) is substantially contributed to the third commonly diagnosed cancer and the second leading cause of cancer-related deaths in the world [1]. Surveillance colonoscopy has been confirmed as a crucial technique to decrease the incidence and mortality of CRC, mainly due to early detection and resection of colorectal adenomas [2–4].

The quality of colonoscopy is the cornerstone of surveillance colonoscopy program, which is associated with several factors, such as bowel preparation, withdrawal time, endoscopists′ experience, and patients' cooperation [5]. Among these, a high-quality bowel preparation is a critically determinant factor to improve adenoma detection rate (ADR) and reduce the incidence of CRC [6]. Inadequate bowel preparation will lead to prolonged procedure duration, decreased visibility of the colonic mucosa, and low ADR [7]. Unfortunately, it is estimated that, up to 25% of patients have poor bowel preparation prior to colonoscopy [8].

Polyethylene glycol (PEG), an orally-administered purgative, is recommended as the first-line bowel preparation regimen, mainly due to its effectiveness and safety [9]. Moreover, several studies have reported that simethicone (SIM), as an antifoaming agent, combined with PEG for bowel preparation can improve bowel preparation quality and patients' tolerance [10,11]. Meanwhile, we discovered that the addition of low-dose SIM (200 mg) to split-dose 2L PEG was as effective as the addition of high-dose SIM (1200 mg) with respect to adequate bowel preparation, ADR, and patients' tolerance in a Chinese population [12]. However, the optimal time of simethicone addition to PEG for bowel preparation remains undetermined. Although Kim H et al. discovered that SIM addition to PEG in the evening of the day prior to colonoscopy improved bowel preparation and increased the diminutive ADR, when compared to the SIM addition to PEG in the morning of colonoscopy, the sample size of this study was as small as less than 80 subjects [13].

Therefore, we performed a prospective randomized controlled trial to determine the optimal time of SIM addition to PEG to improve the quality of bowel preparation and ADR in large samples.

## 2. Methods

### 2.1. Study Design

A prospective, single-center, randomized controlled trial was conducted in Shenzhen People′s Hospital from July 2020 to December 2020. The study protocol was approved by the Human Ethics Committees of the Shenzhen People's Hospital and performed according to the Declaration of Helsinki. All patients were given written informed consent prior to their enrolment. The trial was registered at Chinese Clinical Trial Registry (No. ChiCTR2000033058).

### 2.2. Study Population

Inclusion criteria included consecutive adult participants aged 18–75 years scheduling for outpatient colonoscopies. Exclusion criteria included pregnancy or lactation, heart or kidney failure, suspected allergy to SIM, a history of colon surgery, suspected gastrointestinal obstruction or perforation, a history of adenomatous polyp or CRC, Peutz–Jeghers syndrome (P–J syndrome), familial adenomatous polyposis, and inflammatory bowel disease (IBD).

### 2.3. Randomization and Blinding

A computer-generated randomization table was used to assign eligible subjects: the SIM evening group (SIM 200 mg addition to PEG in the evening of the day prior to colonoscopy) and the SIM morning group (SIM 200 mg addition to PEG in the morning of colonoscopy). The endoscopists were blinded to the randomization process.

### 2.4. Bowel Preparation

Bowel preparation was administered as previously described [12]. In the SIM evening group, the patients were instructed to consume 200 mg SIM (Berlin-Chemie AG, Berlin, Germany) in addition to 1L PEG (Shenzhen Wan he Pharmaceutical Co. Ltd, Shenzhen, China) at 7 PM on the day prior to colonoscopy, and the remaining 1L PEG was ingested in the morning 5 hours before colonoscopy. In the morning group, the patients were instructed to consume 1L PEG at 7 PM on the day prior to colonoscopy, and the 200 mg SIM addition to remaining 1L PEG was ingested in the morning 5 hours before colonoscopy.

### 2.5. Colonoscopy Procedure

All patients were required to fill out a preprocedural questionnaire about preparation compliance before the colonoscopies. All colonoscopies were performed by four experienced endoscopists (>1000 colonoscopies per year) through lower gastrointestinal colonoscopes (CF-HQ190 L/PCF-H190 L, Olympus, Tokyo, Japan) with carbon dioxide insufflation. All colonoscopies were undertaken from 8 AM to 12 AM. All patients received the sedation with midazolam 5 mg and pethidine 50 mg during the procedure.

### 2.6. Data Collection

The occurrence of gastrointestinal symptoms, such as nausea, vomiting, abdominal pain, and bloating, was recorded by an independent researcher prior to colonoscopy. The quality of bowel preparation, cecal intubation time, withdrawal time, and the presence, number, size, and histology of polyps were recorded by endoscopists after the colonoscopy procedure. In order to minimize interobserver variability, all of endoscopists underwent adequate training in the use of Boston Bowel Preparation Scale (BBPS) and Bubble Scale (BS) prior to the start of the study.

### 2.7. Primary Outcomes

The primary outcome was BS score. BS score is based on the amount of foam and bubbles covering the colonic mucosa and quantifies the degree of visual obstruction caused by bubbles and debris according to colonic mucosal visualization. The scores ranged from 0 to 3 and were determined separately for the three segments (right-side colon, transverse colon, and left-side colon) (aupplement materials, Figure S1) [12]. A total score of >6 was considered indicative of successful bowel preparation.

### 2.8. Secondary Outcomes

The second outcome included BBPS score. BBPS score also ranged from 0 to 3 and was determined separately for the three segments (right-side colon, transverse colon, and left-side colon) (supplement materials, Figure S2) [12]. A total score of >6 was considered indicative of successful bowel preparation. Other second outcomes included cecal intubation time, withdrawal time, ADR, polyp detection rate (PDR), adenocarcinoma, adverse events, and patients' tolerance, which were defined as previously described [12].

### 2.9. Sample Size Calculation

In our preclinical trial, we included 50 patients in the SIM evening group and in the SIM morning group, respectively. The percentage of adequate bowel preparation was 96% and 91% in the SIM evening group and in the SIM morning group, respectively. Therefore, a sample size of 181 patients in each group would provide an alpha of 0.005, a power of 90%, and the noninferiority margin of −15% using an online sample size calculator (https://www.cnstat.org/samplesize/12/). Thus, there were at least 208 patients needed in each group by assuming that the missing rate was 15%.

### 2.10. Statistical Analysis

Categorical variables were presented as counts and frequency (%) and compared using the chi-squared test or Fisher's exact test where appropriate. Continuous variables were presented as mean ± standard deviation (SD) or median and interquartile range (IQR) based on the distribution and compared using Student's *t*-test or Mann–Whitney *U* test. Effect sizes are calculated as Cohen standardized mean difference for continuous outcomes. All hypotheses were two-sided, and a *P* value < 0.05 was considered statistically significant.

## 3. Results

### 3.1. Baseline Characteristics

**Figure 1:**
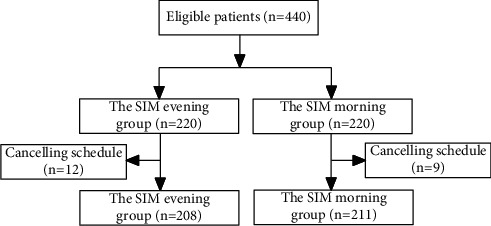
Flow diagram.

A total of 440 patients were eligible and randomly assigned to the SIM evening group and the SIM morning group. Of these patients, 21 patients (twelve patients in the SIM evening group and nine patients in the SIM morning group) were excluded because of personal affairs. Thus, 419 patients were eventually included for further study (Figure 1). There was no significant difference in terms of gender, age, BMI, and indications for colonoscopy between both groups (*P* = 0.30, *P* = 0.92, *P* = 0.52, and *P* = 0.99, respectively) (Table 1).

### 3.2. Quality of Bowel Cleansing

Most of the patients in both groups ingested the overall amount of purgative solution. However, a total of 10 patients (four in the SIM evening group and six in the SIM evening group) ingested less than 90% of the total amount of purgative solution. Therefore, the percentages of the overall purgative solution ingested were comparable between both groups (98.1% vs. 97.2%, *P* = 0.54). There was no significance difference in total BS scores between the two groups (8.76 ± 0.90 vs. 8.65 ± 1.16, effect size 0.13, *P* = 0.28). Nevertheless, the BS scores of the right colon in the SIM evening group were significantly higher than those in the SIM morning group (2.95 ± 0.26 vs. 2.88 ± 0.38, effect side 0.20, *P* = 0.04). The BBPS scores were comparable in terms of the right colon, transverse colon, and right colon comparing the SIM evening group with the SIM morning group (2.80 ± 0.43 vs. 2.72 ± 0.50, effect size 0.18, *P* = 0.07; 2.87 ± 0.36 vs. 2.84 ± 0.43, effect size 0.08, *P* = 0.42; and 2.90 ± 0.33 vs. 2.90 ± 0.36, effect size 0.02, *P* = 0.81, respectively), as well as the total of BBPS scores between the two groups (8.59 ± 0.94 vs. 8.45 ± 1.00, effect size 0.14, *P* = 0.15) (Table 2).

### 3.3. Colonoscopy Findings, Cecal Intubation Time, and Withdrawal Time

Patients in the SIM evening group had a higher ADR than that patients in the SIM morning group; however, there was no statistical significance (34.1% vs. 30.8%, *P* = 0.47). Moreover, the diagnosis of adenocarcinoma, neuroendocrine tumor, hyperplastic polyps, inflammation bowel disease (IBD), and chronic enteritis was comparable comparing the SIM evening group with the SIM morning group (0 vs. 0.9%, *P* = 0.97; 0.5% vs. 0.5%, *P* = 0.499; 9.6% vs. 8.5%, *P* = 0.699; 2.4% vs. 1.9%, *P* = 0.75; and 2.9% vs. 3.3%, *P* = 0.798, respectively). Indeed, the percentage of negative colonoscopy findings in the SIM evening group was similar with that in the SIM morning group (45.7% vs. 51.2%, *P* = 0.259) (Table 3).

A total of 154 adenomas were detected in the SIM evening group, while 153 adenomas were detected in the SIM morning group. According to adenoma location and size stratification, no significant differences were found in the transverse colon and left colon comparing the SIM evening group with the SIM morning group (*P* > 0.05 for all). However, diminutive adenomas (≤5 mm) detected in the right colon were significantly higher in the SIM evening group than in the SIM morning group (62.5% vs. 38.6%, *P* = 0.022) (Table 3).

The cecal intubation time was significantly shorter in the SIM evening group than that in the SIM morning group (3.80 ± 1.81 minutes vs. 4.42 ± 2.03, *P* < 0.001). However, the withdrawal time had no significant difference between the two groups (8.22 ± 2.04 minutes vs. 8.01 ± 2.51 minutes, *P* = 0.094) (Table 3).

### 3.4. Patient Tolerability and Safety

There were no serious adverse events in each group. The percentage of mild adverse events, including nausea, vomiting, abdominal pain, bloating, and sleep disruption were similar in the two groups. Besides, the majority of patients in both groups were willing to ingest the same solution for future colonoscopy procedures (95.7% vs. 95.3%, *P* = 0.84) (Table 4).

## 4. Discussion

Adequate bowel preparation is critical for colonoscopy. However, it has reported that inadequate bowel preparation is as much as up to 25% of patients undergoing colonoscopy [14]. The bowel visibility is one of the most indicators for adequate bowel preparation. SIM is an antifoaming agent, which could not only decrease the formation of bubbles and increase bowel visibility but also reduce the bloating and its associated symptoms [10,11].

Two multicenter randomized trials from the China have found that SIM addition to PEG solution can improve bowel preparation quality, shorten cecal intubation time, and increase ADR [15,16]. A meta-analysis has recently showed that the adjunction of SIM addition to the bowel preparation regimen can significantly improve bowel preparation quality and might increase ADR in settings where low ADR was expected [17]. Besides, we have discovered that low-dose SIM (200 mg) addition to split-dose 2L PEG was as effective as high-dose SIM (1200 mg) addition to split-dose 2L PEG in terms of adequate bowel preparation, ADR, and patients' tolerance in a Chinese population [12]. Therefore, SIM is recommended as an indispensable adjunct for bowel preparation prior to colonoscopy. However, the optimal timing of SIM addition to the bowel preparation regimen remains undetermined. Therefore, a prospective randomized controlled trial involving in a large sample size needed to be performed to confirm the optimal timing of SIM addition to the bowel preparation regimens.

In the present study, we found that there were no significant differences in terms of ADR, BS scores, BBPS scores, withdrawal time, and adverse events comparing the SIM evening group with the SIM morning group. However, we discovered that the SIM evening group was associated with shorter cecal intubation time and higher BS scores in the right colon and diminutive ADR in the right colon, when compared to the SIM morning group.

Matro et al. found that the addition of SIM can improve the bowel preparation quality in the right colon [10]. Moreover, Zhang et al. reported that the addition of SIM can shorten the cecal intubation time and improve the ADR in the right colon [16]. However, Bai et al. found that the addition of SIM can only improve the diminutive ADR [15]. Kim H et al. discovered that SIM addition to PEG in the evening of the day prior to colonoscopy can improve the bowel preparation quality and diminutive ADR than the addition to PEG in the morning of the colonoscopy by a small sample study (*n* = 156) [13]. However, in present study, it is shown that the SIM addition to PEG in the evening of the day prior to colonoscopy was associated with shorter cecal intubation time, higher BS scores in the right colon, and diminutive ADR in the right colon. It has been reported that the brown solid or liquid residues are often found in the right colon, which suggests that the appropriate time of SIM addition is critical for bowel preparation.

There are some strengths of this study compared with the previous study [13]. First, the present study included a larger sample size compared with the previous study (419 vs. 156). Second, it found that the SIM addition to PEG in the evening of the day prior to colonoscopy can shorten the cecal intubation time compared with the SIM addition to PEG in the morning of the day prior to colonoscopy. Third, it revealed that the SIM addition to PEG in the evening of the day prior to colonoscopy can only improve BS scores in right colon and diminutive ADR in the right colon compared with the SIM addition to PEG in the morning of the day prior to colonoscopy. However, there were some limitations. First, this was a single-center study; therefore, a multicenter randomized controlled trial will be conducted to further validate our results. Second, all colonoscopies were undertaken in the morning; therefore, whether the findings of this study will be generalizable for the patients undergoing colonoscopies in the afternoon needed to be further confirmed. Third, although all endoscopists were experienced and trained prior to the start of the study, the effect of interobserver heterogeneity might be not ignored.

In conclusion, the SIM addition to PEG in the evening of the day prior to colonoscopy had the similar ADR, withdrawal time BBPS scores, and adverse events with the SIM addition to PEG in the morning of the colonoscopy. However, the SIM addition to PEG in the evening of the day prior to colonoscopy is associated with shorter cecal intubation time, higher BS scores in the right colon, and higher diminutive ADR in the right colon.

## Figures and Tables

**Table 1:** Baseline characteristics.

	SIM evening group (*n* = 208)	SIM morning group (*n* = 211)	*P* value
*Gender*, *n(%)*			
Male	111 (53.4%)	102 (48.3%)	0.30^a^
Female	97 (46.6%)	109 (51.7%)	
Age (years)	45.0 (34.3–55.0)	44.0 (34.0–56.0)	0.92^b^
BMI (kg/m^2^)	22.3 ± 2.9	22.1 ± 3.7	0.52^c^

*Indications for colonoscopy*, *n (%)*			
Constipation	23 (11.1%)	24 (11.4%)	0.99^a^
Abdominal pain	51 (24.5%)	49 (23.2%)	
Diarrhea	35 (16.8%)	35 (16.6%)	
Bloating	5 (2.4%)	5 (2.4%)	
Screening CRC	94 (45.2%)	98 (46.4%)	

*Note*. BMI, body mass index; CRC, colorectal cancer; a, Pearson's *χ*2 test; *b*, Mann–Whitney test; *c*, Student's *t*-test.

**Table 2:** BS and BBPS scores.

	SIM evening group (*n* = 208)	SIM morning group (*n* = 211)	*P* value	Effect size
*Overall solution intake*, *n (%)*				
Yes	204 (98.1%)	205 (97.2%)	0.54^a^	—
No	4 (1.9)	6 (2.8%)		

*BS scores (mean* *±* *SD)*				
Total scores	8.76 ± 0.90	8.65 ± 1.16	0.28^b^	0.13
Right colon	2.95 ± 0.26	2.88 ± 0.38	**0.04** ^b^	0.20
Transverse colon	2.89 ± 0.36	2.85 ± 0.47	0.38^b^	0.09
Left colon	2.93 ± 0.34	2.92 ± 0.38	0.81^b^	0.03

*BBPS scores (mean* *±* *SD)*				
Total scores	8.59 ± 0.94	8.45 ± 1.00	0.15^b^	0.14
Right colon	2.80 ± 0.43	2.72 ± 0.50	0.07^b^	0.18
Transverse colon	2.87 ± 0.36	2.84 ± 0.43	0.42^b^	0.08
Left colon	2.90 ± 0.33	2.90 ± 0.36	0.81^b^	0.02

*Note*. BS, Bubble Scale; BBPS, Boston Bowel Preparation Scale; SD, standard deviation; a, Pearson's *χ*2 test; *b*, Student's *t*-test.

**Table 3:** Colonoscopy outcomes.

	SIM evening group (*n* = 208)	SIM morning group (*n* = 211)	*P* value
*Diagnosis*, *n (%)*			
Adenoma	71 (34.1%)	65 (30.8%)	0.47^a^
Adenocarcinoma	0 (0%)	2 (0.9%)	0.97^a^
Neuroendocrine tumor	1 (0.5%)	1 (0.5%)	0.499^a^
Hyperplastic polyps	20 (9.6%)	18 (8.5%)	0.699^a^
IBD	5 (2.4%)	4 (1.9%)	0.75^a^
Chronic enteritis	6 (2.9%)	7 (3.3%)	0.798^a^
Normal	95 (45.7%)	108 (51.2%)	0.259^a^
Total adenomas	154	153	—
Right colon	48 (31.2%)	44 (28.8%)	0.62^a^
Transverse colon	41 (26.6%)	47 (30.7%)	0.43^a^
Left colon	65 (42.2%)	62 (40.5%)	0.76^a^

*Right colon*			
≤5 mm	30 (62.5%)	17 (38.6%)	**0.022** ^a^
5–10 mm	11 (22.9%)	15 (34.1%)	0.23^a^
≥10 mm	7 (14.6%)	12 (27.3%)	0.13^a^

*Transverse colon*			
≤5 mm	20 (48.8%)	22 (46.8%)	0.85^a^
5–10 mm	9 (22.0%)	11 (23.4%)	0.87^a^
≥10 mm	12 (29.2%)	14 (29.8%)	0.96^a^

*Left colon*			
≤5 mm	31 (47.7%)	30 (48.4%)	0.94^a^
5–10 mm	21 (32.3%)	22 (35.5%)	0.71^a^
≥10 mm	13 (20.0%)	10 (16.1%)	0.57^a^
Cecal intubation time (minutes)	3.80 ± 1.81	4.42 ± 2.03	<0.001^b^
Withdrawal time (minutes)	8.22 ± 2.04	8.01 ± 2.51	0.094^b^

*Note*. IBD, inflammation bowel disease; a, Bonferroni method *χ*2 test; *b*, Student's *t*-test.

**Table 4:** Patients' compliance and tolerability.

	SIM evening group (*n* = 208)	SIM morning group (*n* = 211)	*P* value
*Tolerability*			
Nausea	6 (2.9%)	7 (3.3%)	0.80^a^
Vomiting	10 (4.8%)	9 (4.3%)	0.79^a^
Abdominal pain	8 (3.8%)	9 (4.3%)	0.83^a^
Bloating	12 (5.8%)	14 (6.6%)	0.71^a^
Sleep disruption	20 (9.6%)	19 (9.0%)	0.83^a^

The same regimen preparation in the future			
Yes	199 (95.7%)	201 (95.3%)	0.84^a^
No	9 (4.3%)	10 (4.7%)	

*Note*. a, Pearson's *χ*2 test.

## Data Availability

The data can be acquired from the corresponding author.

## Abbreviations

SIM: Simethicone
ADR: Adenoma detection rate
BBPS: Boston Bowel Preparation Scale
BS: Bubble Scale
IBD: Inflammatory bowel disease
PEG: Polyethylene glycol
CRC: Colorectal cancer;
PDR: Polyp detection rate
SD: Standard deviation
IQR: Interquartile range.

## Supplementary Materials

Supplementary Materials Figure S1. Bubble scale. A, 0 = bubbles have filled the entire lumen. B, 1 = bubbles have filled 25%–50% luminal diameter. C, 2 = bubbles have filled 5%–25% luminal diameter. *D*, 3 = no or minimal bubbles1. Figure S2. Boston Bowel Preparation Scale (BBPS). A, 0 = colon segment mucosa is not visible. B, 1 = a portion of the colonic mucosa is visible, while other areas are covered by residual stool. C, 2 = a minor amount of residual stool covers some segments of the colonic mucosa; however, other areas are adequately visible. *D*, 3 = colonic mucosa is adequately visible in all segments.
