# Supplementary material for: Optimal Timing of Simethicone Supplement for Bowel Preparation: A Prospective Randomized Controlled Trial
Source: Can J Gastroenterol Hepatol. 2021 Oct 27;2021:4032285. doi: 10.1155/2021/4032285 (PMC8566047; doi:10.1155/2021/4032285)
Supplement: Supplementary Materials — Figure S1. Bubble scale. A, 0 = bubbles have filled the entire lumen. B, 1 = bubbles have filled 25%–50% luminal diameter. C, 2 = bubbles have filled 5%–25% luminal diameter. D, 3 = no or minimal bubbles1. Figure S2. Boston Bowel Preparation Scale (BBPS). A, 0 = colon segment mucosa is not visible. B, 1 = a portion of the colonic mucosa is visible, while other areas are covered by residual stool. C, 2 = a minor amount of residual stool covers some segments of the colonic mucosa; however, other areas are adequately visible. D, 3 = colonic mucosa is adequately visible in all segments. [file 4032285.f1.docx]

**Optimal Timing of Simethicone Supplement for Bowel Preparation: A Prospective Randomized Controlled Trial**

Zhen-wen Wu^1#^, Sheng-gang Zhan^1#^, Mei-feng Yang^2#^, Yi-teng Meng^1^, Feng Xiong^1^, Cheng Wei^1^, Ying-xue Li^1^, Ding-guo Zhang^1^, Zheng-lei Xu^1^, Ben-hua Wu^1^, Rui-yue Shi^1*^, Jun Yao^1*^, Li-sheng Wang^1*^ and De-feng Li^1*^

1. Department of Gastroenterology, Shenzhen People's Hospital (the Second Clinical Medical College, Jinan University; the First Affiliated Hospital, Southern University of Science and Technology), Shenzhen 518020, Guangdong, China
2. Department of Hematology, Yantian District People's Hospital, Shenzhen 518020, Guangdong, China


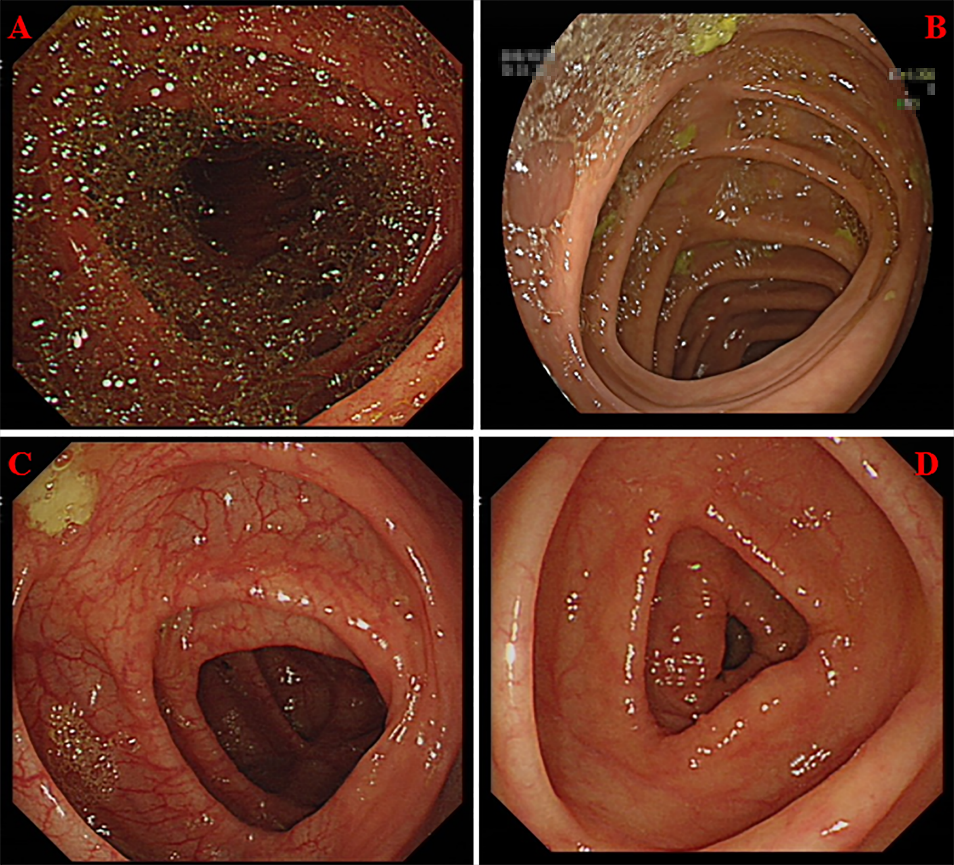


Figure S1. Bubble scale. A, 0 = Bubbles have filled the entire lumen. B, 1 = Bubbles have filled 25%–50% luminal diameter. C, 2 = Bubbles have filled 5%–25% luminal diameter. D, 3 = no or minimal bubbles^1^.


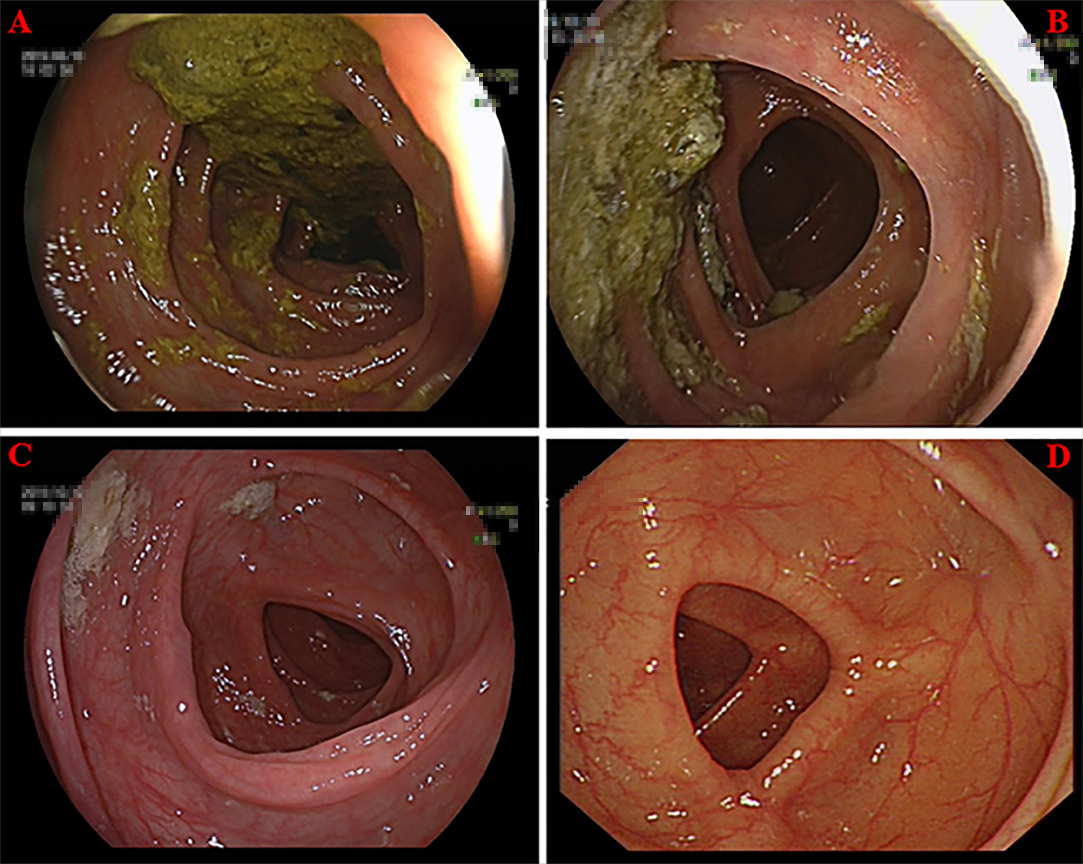


Figure S2. Boston Bowel Preparation Scale (BBPS). A, 0= Colon segment mucosa not visible. B, 1= A portion of colonic mucosa is visible, while other areas are covered by residual stool. C, 2= A minor amount of residual stool covers some segments of the colonic mucosa; however, other areas are adequately visible. D, 3 = Colonic mucosa is adequately visible in all segments^1^.

Reference

1. Li DF, Luo MH, Du QQ, et al. Efficacy of low-dose versus high-dose simethicone with polyethylene glycol for bowel preparation: A prospective randomized controlled trial. J Gastroenterol Hepatol 2020;35:1488-1494.
